# DNA Flap‐Mediated Control of Transcription for Programmable RNA Synthesis

**DOI:** 10.1002/anie.202520198

**Published:** 2026-01-29

**Authors:** Eun Sung Lee, Jisu Woo, Seokjoon Kim, Seok Hyeon Kim, Gun Haeng Lee, Ki Soo Park

**Affiliations:** ^1^ Department of Biological Engineering College of Engineering Konkuk University Seoul Republic of Korea; ^2^ Advanced Materials Program Department of Biological Engineering Konkuk University Seoul Republic of Korea

**Keywords:** T7 promoter engineering, T7 RNA polymerase, transcriptional regulation, DNAzyme, in vitro transcription

## Abstract

The growing success of RNA‐based therapeutics has emphasized the need for precise and programmable RNA synthesis platforms. T7 RNA polymerase (T7RP) is widely utilized for in vitro transcription; however, most existing regulatory strategies rely on auxiliary proteins or chemical modulators. Here, we investigated whether transcription can be regulated solely through nucleic acid sequences. Specifically, we evaluated the effects of single‐stranded DNA flap sequences appended to the 3′ end of the non‐template strand of the T7 promoter, termed the flap promoter, on transcriptional efficiency. Remarkably, we observed a sequence‐dependent inhibitory effect, wherein flaps enriched in pyrimidines (cytosine and thymine) significantly suppressed T7RP‐mediated transcription. Leveraging this intrinsic sequence preference, we developed two novel transcription control platforms, D‐FIT (DNAzyme‐mediated Flap promoter Induced Transcription control) and M‐FIT (MNAzyme‐mediated Flap promoter Induced Transcription control) that enable precise regulation of T7RP activity without the need for auxiliary proteins or chemical agents. These findings uncover a previously unrecognized sequence‐specific regulatory mechanism of T7RP and establish a new framework for the rational design of programmable RNA synthesis systems, with broad potential applications in RNA therapeutics and diagnostics.

## Graphical abstract

Pyrimidine‐rich ssDNA flaps on the T7 promoter inhibit T7 RNA polymerase (T7RP) transcription. Leveraging this pyrimidine bias, we developed DNAzyme‐ and MNAzyme‐mediated flap promoter induced transcription control (D‐FIT and M‐FIT) systems. These platforms enable regulated transcription and sequence‐specific detection, highlighting the programmable potential of T7RP pyrimidine bias.

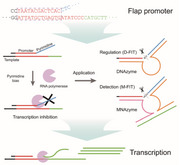

## 1 Introduction

The success of ribonucleic acid (RNA) vaccines during the coronavirus disease 2019 (COVID‐19) pandemic has highlighted the importance of RNA‐based therapeutics and the demand for efficient and programmable RNA synthesis technologies [1, 2, 3]. Among these, T7 RNA polymerase (T7RP)‐mediated transcription has emerged as a widely adopted method, owing to its high transcriptional efficiency and rapid reaction kinetics [4, 5, 6]. It is now a critical tool for in vitro RNA synthesis in various applications, including mRNA vaccine production and RNA therapeutics [7, 8, 9]. In principle, T7RP initiates de novo transcription downstream of a specific promoter sequence comprising 20–23 nucleotides [10, 11]. Although numerous efforts have been made to engineer T7 promoter sequences to enhance transcription efficiency and RNA yield, approaches enabling precise sequence‐dependent control of transcription remain limited [12].

Enzymes are indispensable in biotechnology, finding broad applications in both diagnostics and therapeutics [13, 14]. Recent advances in nucleic acid manipulation (such as modification, degradation, ligation, and amplification) have increased the need for enzyme engineering strategies that modulate catalytic activity [15, 16, 17, 18, 19, 20]. Notably, many nucleic acid enzymes exhibit sequence specificity that governs their functional behavior [21]. For example, the protospacer‐adjacent motif (PAM) recognized by CRISPR‐associated proteins, the T7 promoter sequence recognized by T7RP, and specific restriction sites recognized by restriction endonucleases illustrate how defined sequence motifs can shape enzymatic function [22, 23, 24]. Such intrinsic sequence preferences offer valuable opportunities to fine‐tune enzymatic activity for targeted applications [15, 25, 26].

In this study, we investigated whether T7RP‐mediated transcription could be regulated through specific nucleic acid sequences without the need for chemical modifications or auxiliary regulatory components. We discovered that the presence of a pyrimidine‐rich single‐stranded DNA (ssDNA) flap sequence at the 3′ terminus of the non‐template strand of the T7 promoter significantly suppresses transcription. This finding reveals a previously uncharacterized sequence‐dependent transcriptional inhibition that arises from the intrinsic nucleic acid bias of T7RP. Based on this observation, we developed a novel transcriptional regulation platform utilizing DNAzyme and MNAzyme that enables precise, sequence‐triggered activation of T7RP‐mediated transcription through the removal of inhibitory, pyrimidine‐rich flap structures. By maintaining transcription in a suppressed state prior to flap cleavage, this platform effectively minimizes undesired background transcription and functions as a sequence‐specific transcriptional on/off switch. This strategy expands the current framework for transcriptional control and offers new potential in the development of RNA‐based diagnostics and therapeutics.

## 2 Results and Discussion

### 2.1 Design of Flap Promoter to Regulate Transcription

**FIGURE 1:**
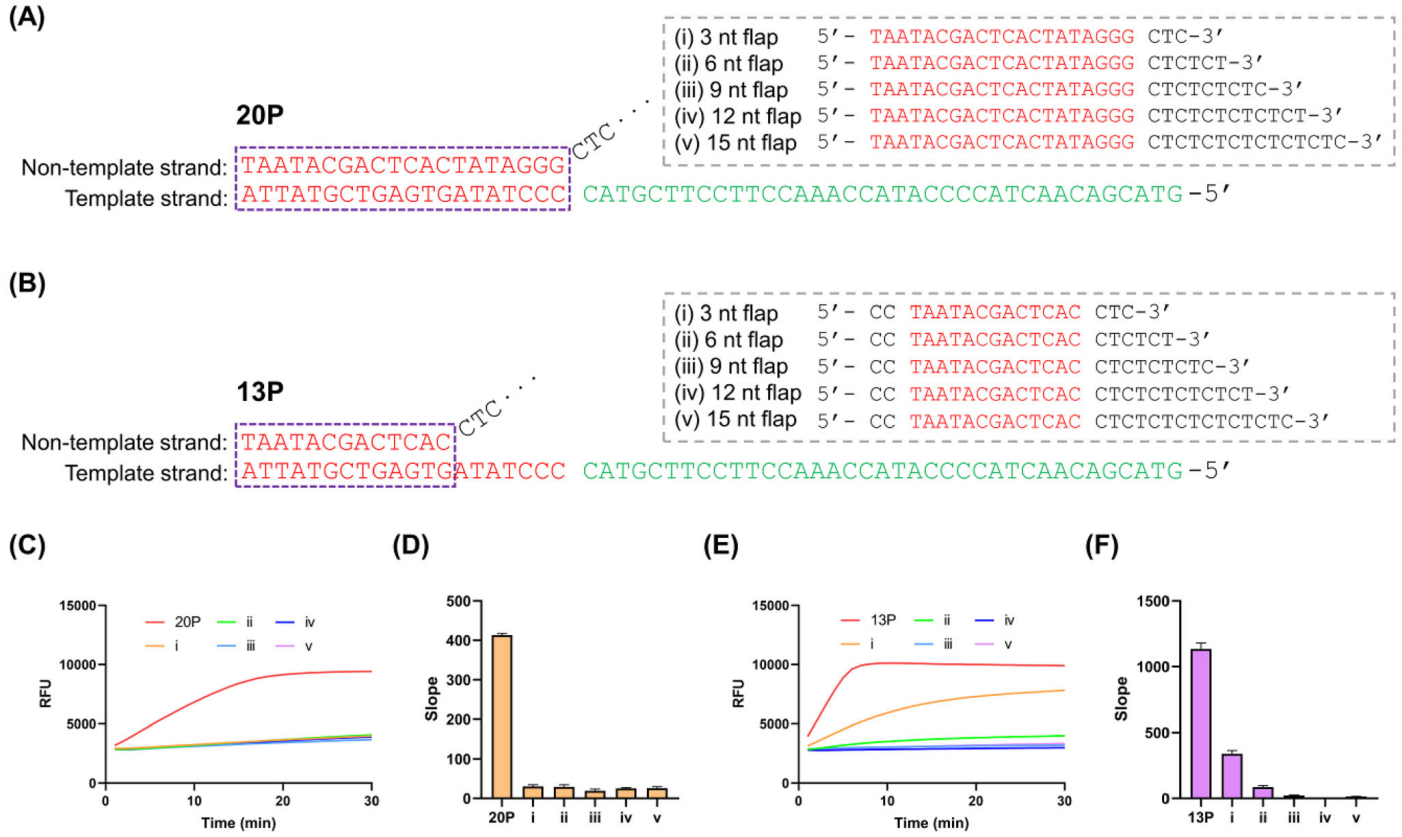
CT flap‐mediated inhibition of T7 promoter‐driven transcription. Nucleotide sequences and schematic representations of (A) the conventional 20‐nucleotide (nt) T7 promoter (20P) and (B) truncated 13‐nt promoter (13P). Each promoter was extended with CT flaps of varying lengths (3–15 nt) and enclosed by a dotted grey box. The promoter region is highlighted in red and enclosed by a dotted violet box, while the transcribed sequence is shown in green. (C) Real‐time transcription kinetics of 20P containing different CT flap lengths. (D) Quantitative analysis of CT flap‐dependent transcriptional inhibition in 20P. (E) Real‐time transcription kinetics of 13P containing different CT flap lengths. (F) Quantitative analysis of CT flap‐dependent transcriptional inhibition in 13P. The slope was calculated as (*RFU_6_* – *RFU_1_*)/5 min, with *RFU_6_* and *RFU_1_* representing fluorescence values at 6 and 1 min in real‐time transcription kinetics. CT, cytosine‐thymine. Data represent mean ± s.d. (n = 3).

T7 promoter‐driven transcription is commonly regulated using Isopropyl β‐D‐1‐thiogalactopyranoside (IPTG)‐inducible systems, riboswitches, or chemical modifications [27, 28, 29, 30]. In this study, we investigated whether T7 transcription could be controlled solely through promoter sequence engineering, without the need for exogenous regulators. We designed a flap ssDNA sequence positioned at the 3′ end of the T7 promoter non‐template strand. In this configuration, only the T7 promoter region was double‐stranded (ds), while the downstream template region remained ssDNA (Figures 1A and 1B).

T7 promoters consist of two functional domains: the recognition region and the initiation region. The initiation region includes an adenine‐thymine (AT)‐rich unwinding segment and a cytosine‐thymine (CT)‐rich transcription start site on the template strand [10]. Based on this structural organization, we extended the 3′ terminus of the non‐template strand with CT repeats to generate flap promoters. We systematically varied the CT flap length and made comparisons between the conventional 20‐nucleotide (nt) promoter (20P) and a truncated 13‐nt variant (13P) (Figure 1) [12]. To improve duplex stability with the template strand, two cytosines were added to the 5′ end of 13P without altering the promoter recognition sequence (Figure 1B) [12, 31]. In the 13P construct, the recognition region remains dsDNA, whereas the downstream unwinding region is presented as ssDNA. This pre‐unwound configuration reduces the energetic barrier for transcription initiation, resulting in higher transcription efficiency compared with the 20P [32, 33, 34].

Gel electrophoresis confirmed the complete hybridization of all flap variants with their template strands, resulting in the formation of double‐stranded promoters (Figure S1). Transcription assays revealed that CT flaps of 3–15 nt markedly inhibited transcription from 20P, whereas in 13P, flaps ≥ 6 nt strongly suppressed activity (Figures 1C–F). These results indicate that CT flaps effectively modulated T7 RNA polymerase activity. Given its higher basal transcriptional activity, the 13P variant was selected for further mechanistic analysis of flap‐mediated transcriptional control.

### 2.2 Assessment of T7 RNA Polymerase Nucleotide Preference

**FIGURE 2:**
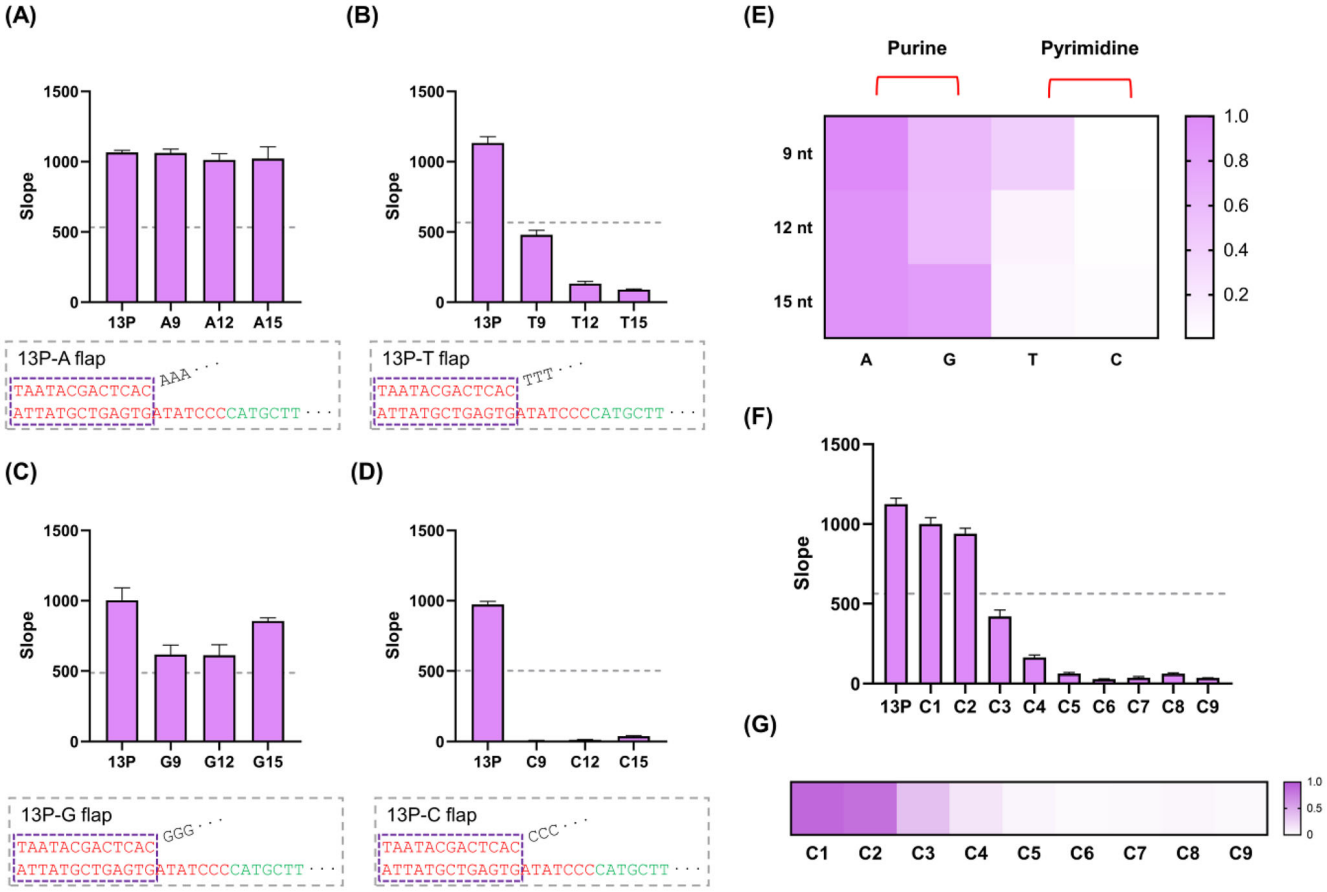
Transcriptional inhibition of the 13P by nucleotide‐specific flaps. (A–D) Transcriptional activity of 13P variants containing 9, 12, or 15 nt flaps composed exclusively of A, T, C, or G. The promoter region is highlighted in red and enclosed by a dotted violet box, while the transcribed sequence is shown in green. The grey dotted box denotes the sequence of each flap promoter structure. (E) Heatmap representation of transcriptional slopes from (A–D), normalized to the 13P control. (F) Transcriptional activity of C flap variants ranging from 1 to 9 nt (C1–C9). (G) Heatmap representation of transcriptional slopes from (F). nt, nucleotide; 13P, 13‐nt promoter; A, adenine; T, thymine; C, cytosine; G, guanine. The grey dotted line indicates 50% of the slope value of the 13P control. Data represent mean ± s.d. (n = 3).

To examine whether the flap sequence composition influences transcriptional inhibition, we designed flap promoters composed exclusively of adenine (A), thymine (T), cytosine (C), or guanine (G) at lengths of 9, 12, or 15 nt and evaluated their transcriptional activity. Pyrimidine‐based flaps (C and T) inhibited transcription by more than 50%, with cytosine flaps showing the strongest effect. In contrast, purine‐based flaps (A and G) did not reduce activity by more than 50% (Figures 2A–E). Gel electrophoresis confirmed that all A, T, C, G flap promoters successfully hybridized with their template strands to form ds T7 promoters (Figure S2). These results are in parallel with the inhibitory effects observed with CT flaps (Figure 1), suggesting that T7RP exhibits a sequence‐dependent preference against pyrimidines on the non‐template strand.

Given that the transcription start site of the T7 promoter is intrinsically pyrimidine‐rich on the template strand, these findings indicate that T7RP exhibits strong affinity toward template‐strand pyrimidines, while interacting less favorably with pyrimidines on the non‐template strand. To further define the inhibitory threshold, we generated cytosine flaps ranging from 1 to 9 nt. Gel electrophoresis confirmed proper hybridization for all C flap variants (Figure S3). Transcription assays revealed that even a 3 nt C flap reduced activity by over 50% (Figures 2F and 2G), indicating that minimal pyrimidine extensions on the non‐template strand can substantially suppress T7 transcription. This inhibition was comparable to that observed with CT flaps starting at 3 nt (Figure 1F).

While sequences of identical nucleotides can achieve strong inhibition, they pose synthetic challenges and may limit design flexibility [35]. In contrast, flaps incorporating two or more alternating nucleotides enable broader sequence customization. Based on these considerations, we selected the 12 nt CT flap (CT12) as the optimal candidate for use in subsequent studies.

### 2.3 Validation of Various Flap Promoter Structures

**FIGURE 3:**
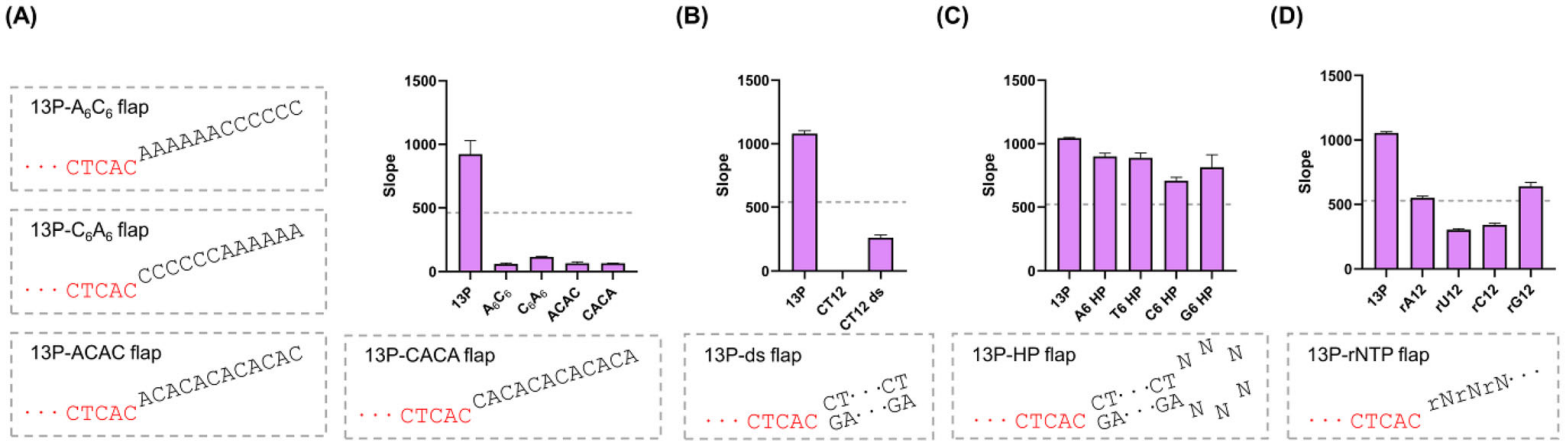
Evaluation of various flap structures. (A) Transcriptional activity of 12 nt flaps containing A and C. (B) Transcriptional activity of single‐stranded and double‐stranded 12 nt CT flaps. (C) Transcriptional activity of hairpin (HP) flaps with different loop sequences. N denotes a DNA nucleotide (A, T, C, or G). (D) Transcriptional activity of RNA flaps. rN denotes an RNA nucleotide (rA, rU, rC, or rG). The promoter region is highlighted in red, and the grey dotted boxes denote the sequence of respective flap promoter structures. nt, nucleotide; ds, double‐stranded; 13P, 13‐nt promoter; A, adenine; T, thymine; C, cytosine; G, guanine; U, uracil; rNTP, ribonucleoside triphosphate. The grey dotted line indicates 50% of the slope value of the 13P control. Data represent mean ± s.d. (n = 3).

To determine whether purine residues could mitigate pyrimidine‐induced transcriptional inhibition, we designed 12 nt flap sequences containing equal numbers of A. Four configurations were tested: A_6_C_6_, C_6_A_6_, alternating AC repeats (ACAC…), and CA repeats (CACA…) (Figures 3A and S4). All variants hybridized efficiently to the template strand. However, transcriptional inhibition persisted, indicating that the dominant inhibitory effect of C could not be overcome by the inclusion of A.

We next investigated whether transcriptional regulation could be modulated by altering flap structure. We generated a ds flap by annealing 12 nt complementary sequences, and hairpin (HP) flaps were designed with 12 nt stem sequences and a 6 nt loop composed of either A, T, C, or G (Figures 3B and 3C). In ds flap, transcriptional activity showed partial recovery, but it was still inhibited by 76% (Figures 3B, S5A and S5B). HP flaps with A, T, or G loops did not reduce activity, but activity was comparable with that of 13P. HP flaps with the C loop provided a 32% transcription inhibition effect (Figures 3C, S5C, and S5D), and this exception might be due to the cytosine preference of T7RP.

Finally, we evaluated RNA flap sequences to assess their impact on transcription. All four RNA flap variants (rA, rU, rC, and rG) reduced transcription, with pyrimidine‐based RNA flaps (rU and rC) exhibiting stronger inhibition than purine‐based flaps (rA and rG) (Figures 3D and S6). The observed suppression may be linked to the DNA‐dependent nature of T7RP, which could influence its interaction with RNA flaps.

### 2.4 DNAzyme‐Mediated Flap Promoter Induced Transcription Control (D‐FIT)

**FIGURE 4:**
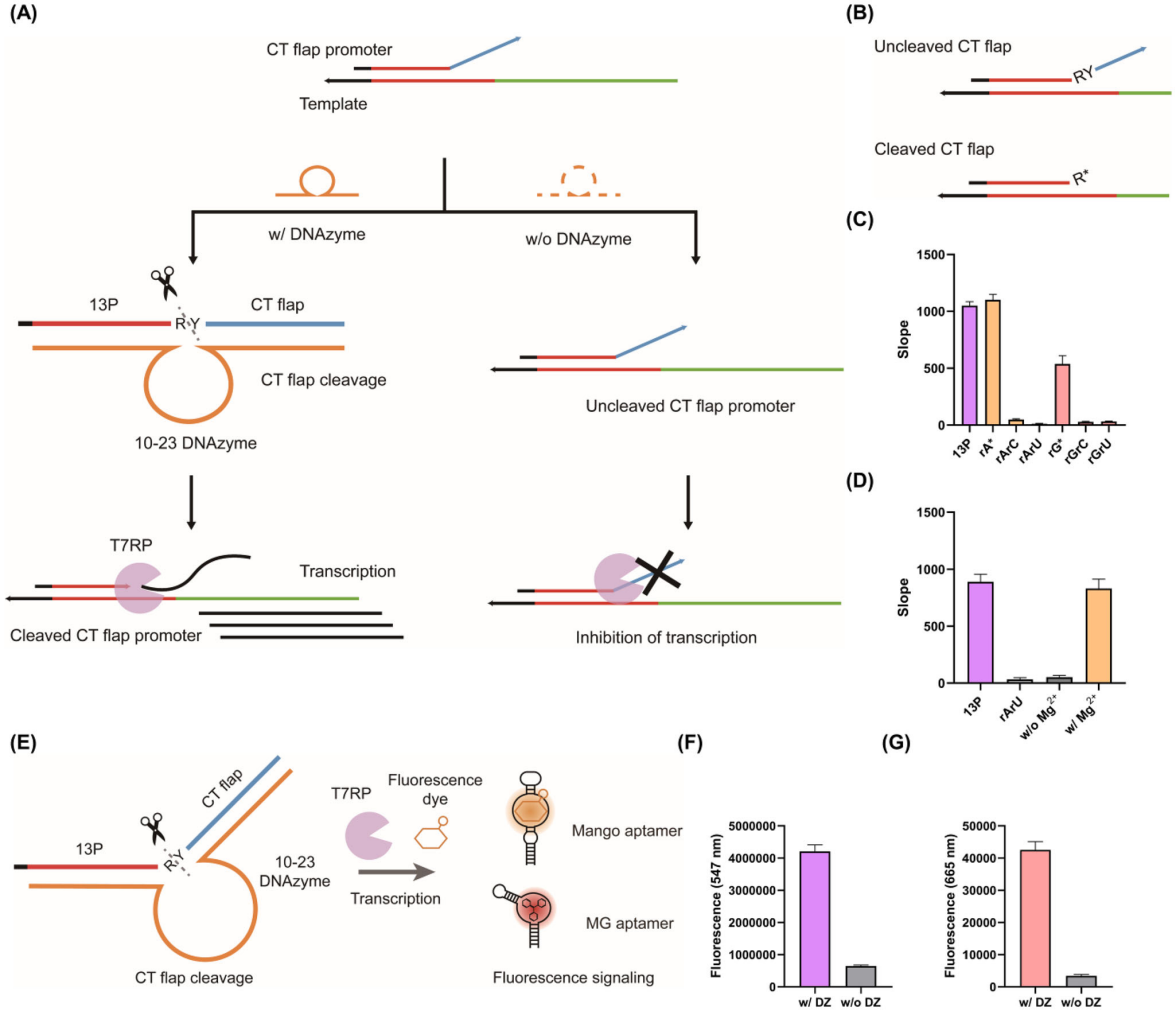
Regulation of T7 transcriptional activity by the DNAzyme‐mediated flap promoter induced transcription control (D‐FIT) system. (A) Schematic overview of the D‐FIT system. (B) Illustration of the cleaved promoter structure in the D‐FIT system. (C) Optimization of the cleavage site sequence for the D‐FIT cleavage system. (D) Assessment of Mg^2+^ dependency in the D‐FIT system. (E) Schematic illustration of light‐up aptamer signaling using the D‐FIT system. (F) Validation of Mango aptamer transcription controlled by the D‐FIT system. (G) Validation of malachite green (MG) aptamer transcription controlled by the D‐FIT system. RY sequence (R = rA or rG; Y = rU or rC). T7RP, T7 RNA polymerase; CT, cytosine‐thymine; 13P, 13‐nucleotide promoter; DZ, DNAzyme; A, adenine; U, uracil; C, cytosine; G, guanine. Data represent mean ± s.d. (n = 3).

Given the strong inhibitory effect of pyrimidine‐rich flaps, we developed a DNAzyme‐mediated flap promoter induced transcription control (D‐FIT) system to restore transcription by site‐specific flap cleavage (Figure 4A). The system uses a 10–23 DNAzyme targeting an RY sequence (R = rA or rG; Y = rU or rC) inserted between the 13P and CT12 flap regions. Cleavage at this defined site removes the CT12 flap, thereby relieving transcriptional repression and enabling transcriptional recovery. DNAzymes were selected because their programmable binding arms allow precise placement of the cleavage site without the need for fixed recognition sequences, which would otherwise constrain system design [36]. Among reported DNAzymes, the widely used 10–23 DNAzyme was chosen due to its broad substrate compatibility and flexibility in cleavage‐site selection, making it particularly well suited for the current flap promoter architecture [37, 38, 39].

We first tested four RY combinations (rArC, rArU, rGrC, and rGrU) to determine whether transcriptional inhibition was retained and whether recovery depended on the 3′ terminal residue after cleavage. All combinations strongly suppressed transcription (Figures 4B, 4C and S7). Following DNAzyme cleavage, full recovery occurred with rA‐terminated promoters (rA*), whereas rG‐terminated promoters (rG*) showed partial recovery (51%). Based on these results, the rArU flap was chosen as the optimal candidate, combining strong inhibition with efficient recovery.

Because Mg^2+^ is essential for 10–23 DNAzyme activity, we next examined its effect on the D‐FIT system. In the absence of Mg^2+^, flap cleavage was abolished, and transcription remained inhibited, whereas Mg^2+^ addition restored both cleavage and transcriptional activity (Figures 4D and S8).

To optimize cleavage efficiency, we used fluorescein–quencher (F–Q) flap probes labelled with 5′‐FAM and 3′‐BHQ‐1. Fluorescence measurements showed that cleavage peaked at 40 min (Figure S9A). Varying the DNAzyme‐to‐flap promoter ratio revealed that a 1:4 ratio maximized normalized cleavage efficiency without significantly interfering with transcription (Figure S9B). Optimization of MgCl_2_ concentration showed maximal cleavage at 25 mM, whereas higher concentrations (50 mM) reduced activity (Figure S9C), likely due to hindered dissociation of cleaved products from the DNAzyme [39, 40].

Finally, we validated RNA synthesis accuracy using a light‐up aptamer assay (Figure 4E). The template strand encoding Mango (TO1‐biotin binding) or Malachite Green (MG binding) aptamers produced fluorescence only when the rArU flap was cleaved. These results demonstrate that D‐FIT enables cleavage‐dependent transcription with accurate RNA product generation (Figures 4F and 4G) [23]. Collectively, these findings indicate that D‐FIT functions as a modular transcriptional switch in which transcription is maintained in a suppressed state by the CT12 flap and is selectively activated upon DNAzyme‐mediated flap cleavage.

### 2.5 MNAzyme‐Mediated Flap Promoter Induced Transcription Control (M‐FIT)

**FIGURE 5:**
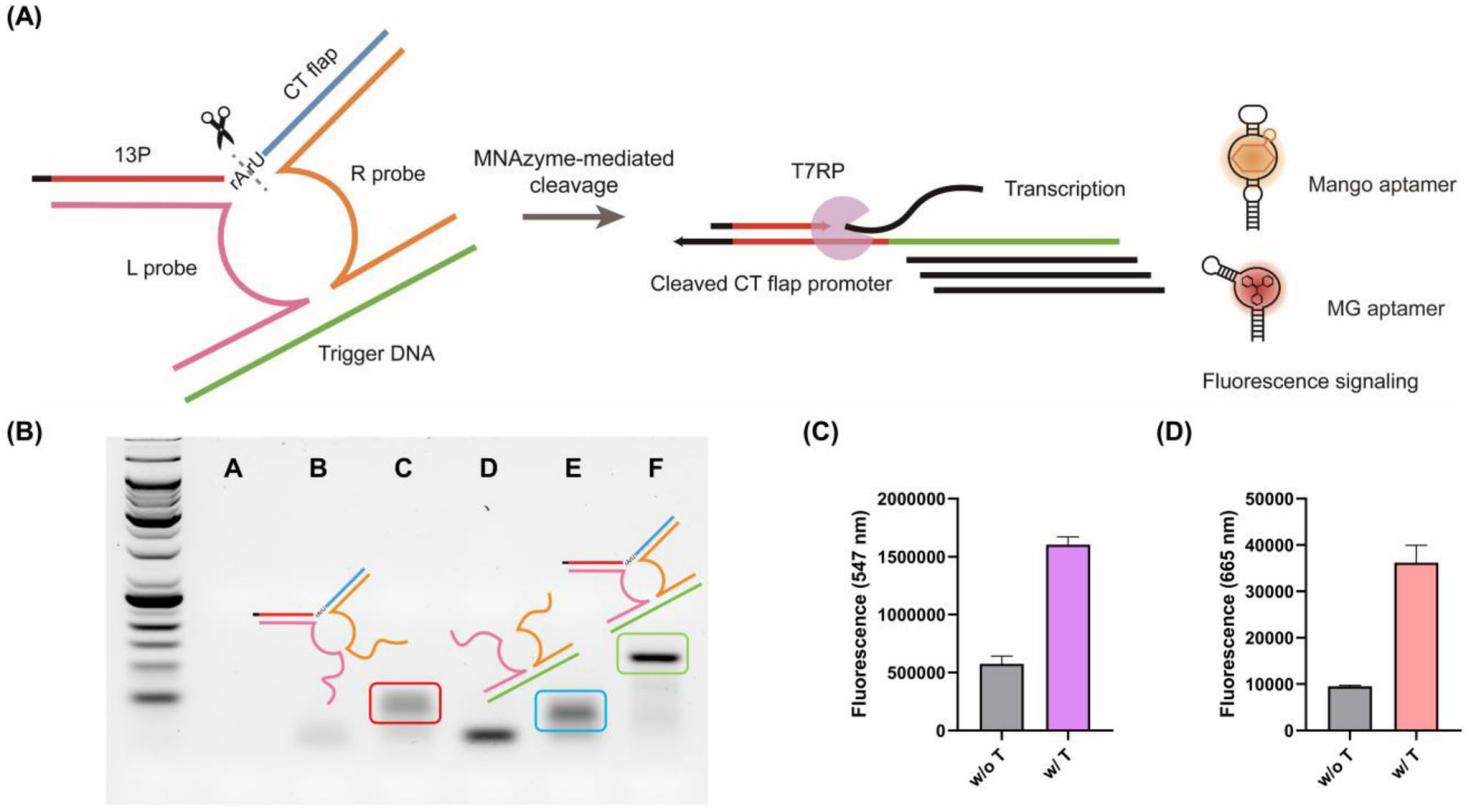
Regulation of transcriptional activity by the MNAzyme‐mediated flap promoter induced transcription control (M‐FIT) system. (A) Mechanism of light‐up aptamer signaling through the M‐FIT system. (B) Confirmation of MNAzyme complex formation by agarose gel electrophoresis. Lane A: F; lane B: F + T; lane C: F + L + R; lane D: L + R; lane E: L + R + T; lane F: F + L + R + T. Bands marked with colored boxes correspond to the DNA structures shown above each box. (C) Validation of Mango aptamer transcription control by the M‐FIT system. (D) Validation of malachite green (MG) aptamer transcription control by the M‐FIT system. 13P, 13‐nucleotide promoter; T7RP, T7 RNA polymerase; F, rArU flap; L, L probe; R, R probe; T, trigger DNA. Data represent mean ± s.d. (n = 3).

To enable target‐specific activation, we adapted the D‐FIT design to a multi‐component nucleic acid enzyme (MNAzyme) format, producing the MNAzyme‐mediated flap promoter induced transcription control (M‐FIT) system (Figure 5A). In this system, the 10–23 DNAzyme catalytic core is split into two fragments (8/7 configuration) that assemble only in the presence of a specific trigger DNA. The trigger bridges the L probe, R probe, and rArU flap promoter, thereby reconstituting the catalytic core and enabling flap cleavage.

Gel electrophoresis confirmed functional MNAzyme assembly only in the presence of trigger DNA (Figure 5B). Transcription assays showed reduced activity in the uncleaved state, with recovery following trigger DNA‐dependent flap cleavage (Figure S10). Similarly, Mango and MG aptamer fluorescence was detected only when trigger DNA was present, confirming target‐dependent transcription activation (Figures 5C and 5D).

These results demonstrate that M‐FIT enables sequence‐specific, trigger DNA‐dependent transcriptional activation, producing RNA output signals only upon target recognition. This regulatory behavior arises from the intrinsic nucleotide bias of T7RP and the conditional assembly of the MNAzyme complex, which forms exclusively in the presence of a trigger DNA. As a result, flap cleavage and subsequent transcription initiation occur only under sequence‐matched conditions.

Given this target‐dependent activation mechanism, M‐FIT represents a promising framework for RNA production and diagnostic applications responsive to nucleic acids. In particular, DNA products generated through isothermal amplification methods, such as recombinase polymerase amplification (RPA) or loop‐mediated isothermal amplification (LAMP), could be integrated with the M‐FIT system to trigger transcription of a light‐up RNA aptamer, thereby providing a fluorescence readout [41, 42, 43].

## 3 Conclusion

In this study, we demonstrated that T7 promoter‐driven transcription can be precisely regulated by embedding defined nucleic acid sequences into the promoter region, without the need for chemical modifications. Specifically, incorporation of a pyrimidine‐rich ssDNA flap at the 3′ terminus of the non‐template strand significantly suppressed transcription, revealing a strong sequence bias of T7 RNA polymerase toward pyrimidine nucleotides. Among these, cytosine exerted the strongest inhibitory effect, leading to the identification of a 12 nt alternating cytosine‐thymine sequence (CT12) as the optimal inhibitory element for downstream applications.

Building on this principle, we engineered two protein‐free, nucleic acid‐mediated transcription control platforms: the DNAzyme‐based D‐FIT and its MNAzyme‐based counterpart, M‐FIT. Both systems enabled sequence‐specific cleavage of the inhibitory flap, as validated by real‐time fluorescence monitoring and light‐up RNA aptamer assays. Notably, the M‐FIT platform allowed conditional transcriptional activation in response to a designated trigger DNA sequence, demonstrating its utility for programmable, sequence‐specific RNA synthesis.

Together, these findings establish flap promoter‐based designs as a versatile framework for regulated RNA production and provide a promising foundation for the development of nucleic acid‐driven diagnostic technologies. The present study was conducted using an in vitro transcription system with synthetic DNA templates. Therefore, further validation using longer templates, such as full‐length mRNA, will be necessary. In addition, future studies should aim to elucidate the molecular basis underlying the pyrimidine‐rich flap bias observed in T7RP‐mediated transcription and to investigate whether transcriptional upregulation beyond basal promoter activity can be achieved through the rational design of purine‐rich or other engineered flap structures.

## Conflicts of Interest

The authors declare no conflict of interest.

## Supporting information




**Supporting File 1**: anie71338‐sup‐0001‐SuppMat.docx.

## Data Availability

The data that support the findings of this study are available in the supplementary material of this article.
