# Supplementary material for: DNA Flap‐Mediated Control of Transcription for Programmable RNA Synthesis
Source: Angew Chem Int Ed Engl. 2026 Jan 29;65(18):e20198. doi: 10.1002/anie.202520198 (PMC13110767; doi:10.1002/anie.202520198)
Supplement: Supplementary file 1 — Supporting File 1: anie71338‐sup‐0001‐SuppMat.docx. [file ANIE-65-e20198-s001.docx]

Supporting Information for

DNA Flap-Mediated Control of Transcription for Programmable RNA Synthesis

Eun Sung Lee,^+[b]^ Jisu Woo,^+[b]^ Seokjoon Kim,^[b]^ Seok Hyeon Kim,^[b]^ Gun Haeng Lee,^[b]^ and Ki Soo Park*^[a]^

[a] Prof. Dr. K. S. Park
Department of Biological Engineering, College of Engineering, Konkuk University, Seoul 05029, Republic of Korea

Advanced Materials Program, Department of Biological Engineering, Konkuk University, Seoul 05029, Republic of Korea
E-mail: [akdong486@konkuk.ac.kr](mailto:akdong486@konkuk.ac.kr)

[b] Dr. E. S. Lee; J. Woo; Dr, S. Kim; S. H. Kim; G. H. Lee
Department of Biological Engineering
College of Engineering, Konkuk University
Seoul 05029, Republic of Korea

^+^ These authors contributed equally to this study

**Experimental section**

**Real-time monitoring of T7 RNA polymerase-mediated transcription**

All oligonucleotides used in this study (Table S1-3) were synthesized and purchased from Integrated DNA Technologies (Coralville, IA, USA). T7 RNA polymerase (T7RP)-mediated transcription was monitored using a CFX96 qPCR system (Bio-Rad Laboratories; Hercules, CA, USA). Real-time amplification curves were analyzed to assess transcriptional activity. The reaction mixture (19 µL final volume) contained 2 µL of 10X T7RP buffer (Enzynomics; Daejeon, Republic of Korea), 4 µL of 2.5 mM ribonucleoside triphosphates (rNTPs; New England Biolabs, Ipswich, MA, USA), 1 µL of 1 µM template strand, 2 µL of 10X SYBR Green II (Invitrogen; Carlsbad, CA, USA), 0.4 µL of T7RP (Enzynomics), 0.4 µL of RNase inhibitor (Enzynomics), and diethyl pyrocarbonate (DEPC)-treated water to volume. Additionally, 1 µL of 1 µM flap promoter was placed on the inner lid of each tube to start transcription simultaneously across all samples. The tubes were briefly centrifuged to mix before starting the reaction. The reaction was performed at 37 °C, with fluorescence signals acquired every minute. The initial transcription rate was calculated from the slope of the fluorescence increase over time, defined as: Y = (RFU₆ - RFU₁) / 5, where RFU represents the relative fluorescence units measured at the indicated time points.

**Agarose gel electrophoresis**

Agarose gel electrophoresis was conducted to confirm hybridization between the flap promoters and the template strand. Each nucleic acid probe was prepared at 1 µM and loaded onto a 2.5% agarose gel. Electrophoresis was conducted at 135 V for 35 min, and gels were imaged using a ChemiDoc Imaging System (Bio-Rad Laboratories).

**DNAzyme and MNAzyme-mediated cleavage**

Catalytic activities of DNAzyme and MNAzyme were assessed in 20 µL reaction mixtures containing 2 µL of 100 mM Tris-acetate buffer (pH 8.0), 2 µL of MgCl_2_, 1 µL of DNA probes, and DEPC-treated water. Concentrations of DNA probes and MgCl_2_ were varied according to experimental conditions. For real-time fluorescence monitoring, 2 µL of DNAzyme or MNAzyme reaction products were analyzed under the conditions described below, with the flap promoter concentration fixed at 50 nM. For light-up RNA aptamer assays, 5 µL of reaction products were used, with the flap promoter concentration maintained at 20 nM.

**Denaturing Urea Polyacrylamide Gel Electrophoresis (Urea-PAGE)**

Denaturing urea-PAGE was conducted to verify flap promoter cleavage mediated by DNAzyme and MNAzyme. The nucleic acid probes were mixed 1:1 with 2X NOVEX UREA PAGE loading dye (Invitrogen), heat-denatured at 95°C for 5 min and immediately chilled on ice to prevent rehybridization. Samples were run on a 14% urea gel pre-run at 200 V for 40 min, followed by electrophoresis at 150 V for 50 min. Gels were stained with GreenStar Nucleic Acid Staining Solution (Bioneer; Daejeon, Republic of Korea) for 10 min in the dark and imaged with a ChemiDoc imaging system (Bio-Rad Laboratories).

**Light-up RNA aptamer assay**

Transcription regulation by the flap promoter was monitored using two light-up RNA aptamers, Mango and Malachite Green (MG). Each 50 µL transcription reaction contained 5 µL of flap promoter, 5 µL of 10X T7RP buffer, 10 µL of 2.5 mM rNTPs, 1 µL of 1 µM templated strand, 10 µL of either TO1-biotin (1 µM; Applied Biological Materials; Richmond, Canada) or MG (25 µM; Sigma-Aldrich; St. Louis, MO, USA), 0.4 µL of T7RP, and 0.4 µL of RNase inhibitor, with DEPC-treated water added to the final volume. Reactions were incubated at 37 °C for 10 min and then transferred to a 384-well plate (SPL Life Science; Pocheon, Republic of Korea) for fluorescence measurement. Fluorescence was measured using a SpectraMax iD5 microplate reader (Molecular Devices; San Jose, CA, USA) with excitation/emission wavelengths of 507/547 nm for TO1-biotin and 616/665 nm for MG.

**Table S1.** DNA sequences used in this study

| **DNA probe** | **Sequence (5′→3′)** |
| --- | --- |
| **Fig. 1, Fig. S1** | |
| Template strand | GTACGACAACTACCCCATACCAAACCTTCCTTCGTACCCCTATAGTGAGTCGTATTAGGAAGGAGGG |
| 20P | TAATACGACTCACTATAGGG |
| 20P CT3 | TAATACGACTCACTATAGGGCTC |
| 20P CT6 | TAATACGACTCACTATAGGGCTCTCT |
| 20P CT9 | TAATACGACTCACTATAGGGCTCTCTCTC |
| 20P CT12 | TAATACGACTCACTATAGGGCTCTCTCTCTCT |
| 20P CT15 | TAATACGACTCACTATAGGGCTCTCTCTCTCTCTC |
| 13P | CCTAATACGACTCAC |
| 13P CT3 | CCTAATACGACTCACCTC |
| 13P CT6 | CCTAATACGACTCACCTCTCT |
| 13P CT9 | CCTAATACGACTCACCTCTCTCTC |
| 13P CT12 | CCTAATACGACTCACCTCTCTCTCTCT |
| 13P CT15 | CCTAATACGACTCACCTCTCTCTCTCTCTC |
| **Fig. 2A-E, Fig. S2** | |
| 13P A9 | CCTAATACGACTCACAAAAAAAAA |
| 13P A12 | CCTAATACGACTCACAAAAAAAAAAAA |
| 13P A15 | CCTAATACGACTCACAAAAAAAAAAAAAAA |
| 13P T9 | CCTAATACGACTCACTTTTTTTTT |
| 13P T12 | CCTAATACGACTCACTTTTTTTTTTTT |
| 13P T15 | CCTAATACGACTCACTTTTTTTTTTTTTTT |
| 13P C9 | CCTAATACGACTCACCCCCCCCCC |
| 13P C12 | CCTAATACGACTCACCCCCCCCCCCCC |
| 13P C15 | CCTAATACGACTCACCCCCCCCCCCCCCCC |
| 13P G9 | CCTAATACGACTCACGGGGGGGGG |
| 13P G12 | CCTAATACGACTCACGGGGGGGGGGGG |
| 13P G15 | CCTAATACGACTCACGGGGGGGGGGGGGGG |
| **Fig. 2F and 2G, Fig. S3** | |
| 13P C1 | CCTAATACGACTCACC |
| 13P C2 | CCTAATACGACTCACCC |
| 13P C3 | CCTAATACGACTCACCCC |
| 13P C4 | CCTAATACGACTCACCCCC |
| 13P C5 | CCTAATACGACTCACCCCCC |
| 13P C6 | CCTAATACGACTCACCCCCCC |
| 13P C7 | CCTAATACGACTCACCCCCCCC |
| 13P C8 | CCTAATACGACTCACCCCCCCCC |
| 13P C9 | CCTAATACGACTCACCCCCCCCCC |
| **Fig. 3A, Fig. S4** | |
| 13P A_6_C_6_ | CCTAATACGACTCACAAAAAACCCCCC |
| 13P C_6_A_6_ | CCTAATACGACTCACCCCCCCAAAAAA |
| 13P ACAC | CCTAATACGACTCACACACACACACAC |
| 13P CACA | CCTAATACGACTCACCACACACACACA |
| **Fig. 3B, Fig. S5A and S5B** | |
| 13P ds | TGTGTGTGTGTG |
| **Fig. 3C, Fig. S5C and S5D** | |
| 13P HP(A) | CCTAATACGACTCACCTCTCTCTCTCTAAAAAAAGAGAGAGAGAG |
| 13P HP(T) | CCTAATACGACTCACCTCTCTCTCTCTTTTTTTAGAGAGAGAGAG |
| 13P HP(C) | CCTAATACGACTCACCTCTCTCTCTCTCCCCCCAGAGAGAGAGAG |
| 13P HP(G) | CCTAATACGACTCACCTCTCTCTCTCTGGGGGGAGAGAGAGAGAG |
| **Fig. 3D, Fig S6** | |
| 13P rA12 | CCTAATACGACTCACrArArArArArArArArArArArA |
| 13P rU12 | CCTAATACGACTCACrUrUrUrUrUrUrUrUrUrUrUrU |
| 13P rC12 | CCTAATACGACTCACrCrCrCrCrCrCrCrCrCrCrCrC |
| 13P rG12 | CCTAATACGACTCACrGrGrGrGrGrGrGrGrGrGrGrG |

The red, orange, blue and brown colors represent the T7 promoter, flap, hairpin loop region, and flap complementary sequence. 20P, 13P, A, T, C, G, U, and HP represent 20-nucleotide promoter, 13-nucleotide promoter, adenine, thymine, cytosine, guanin, uracil, and hairpin, respectively.

**Table S2.** DNA sequences used in the D-FIT system

| **DNA probe** | **Sequence (5′→3′)** |
| --- | --- |
| Template strand (Mango) | GTACGACAACTACCCCATACCAAACCTTCCTTCGTACCCCTATAGTGAGTCGTATTAGGAAGGAGGG |
| Template strand (MG) | GGATCCATTCGTTACCTGGCTCTCGCCAGTCGGGATCCCCCTATAGTGAGTCGTATTAGGAAGGAGGG |
| DNAzyme (rA) | AGAGAGAGGGGCTAGCTACAACGAGTGAGTCGT |
| rA* | CCTAATACGACTCACrA |
| rArC | CCTAATACGACTCACrArCCTCTCTCTCTCT |
| rArU | CCTAATACGACTCACrArUCTCTCTCTCTCT |
| DNAzyme (rG) | AGAGAGAGAGGCTAGCTACAACGAGTGAGTCGT |
| rG* | CCTAATACGACTCACrG |
| rGrC | CCTAATACGACTCACrGrCCTCTCTCTCTCT |
| rGrU | CCTAATACGACTCACrGrUCTCTCTCTCTCT |
| rArU F-Q | FAM-CCTAATACGACTCACrArUCTCTCTCTCTCT-BHQ-1 |

The red and orange colors represent the T7 promoter and flap region, respectively. MG, A, T, C, G, and U represent malachite green, adenine, thymine, cytosine, guanin, and uracil, respectively.

**Table S3*.*** DNA sequences used in the M-FIT system

| **DNA probe** | **Sequence (5′→3′)** |
| --- | --- |
| Template strand (mango) | GTACGACAACTACCCCATACCAAACCTTCCTTCGTACCCCTATAGTGAGTCGTATTAGGAAGGAGGG |
| Template strand (MG) | GGATCCATTCGTTACCTGGCTCTCGCCAGTCGGGATCCCCCTATAGTGAGTCGTATTAGGAAGGAGGG |
| rArU | CCTAATACGACTCACrArUCTCTCTCTCTCT |
| R probe | AGAGAGAGAGGCTAGCTCTGATAAGCTA |
| L probe | TCAACATCAGTACAACGAGTGAGTCGT |
| Trigger DNA | TAGCTTATCAGACTGATGTTGA |

The red and orange colors represent the T7 promoter and flap region, respectively. MG, A, T, C, G, and U represent malachite green, adenine, thymine, cytosine, guanin, and uracil, respectively


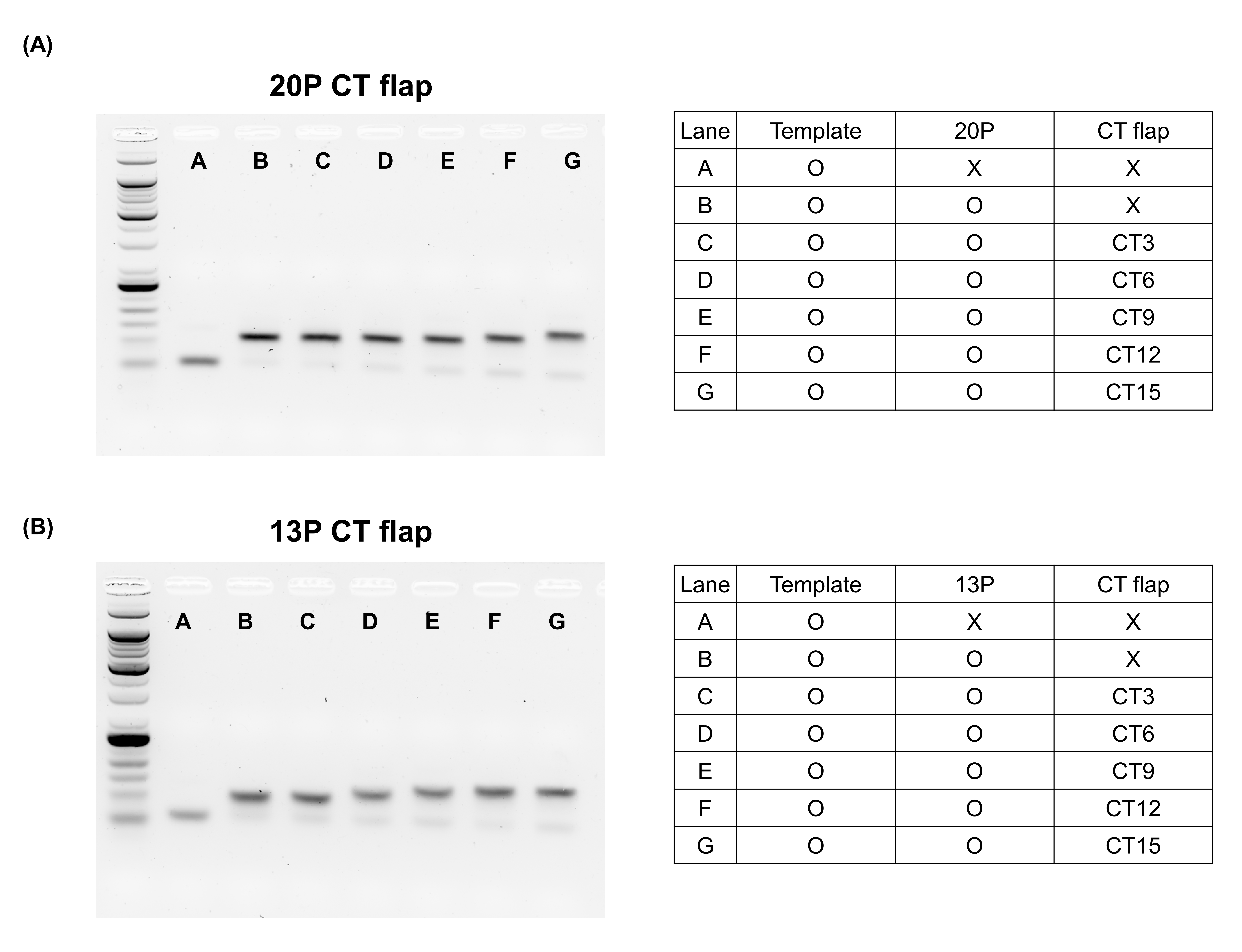


**Figure S1*.***  Confirmation of DNA probe hybridization by 2.5% agarose gel electrophoresis. (A) Hybridization between the transcription template strand and 20P CT flaps. The table on the right shows the probe combinations loaded in each lane. (B) Hybridization between the transcription template strand and 13P CT flaps. The table on the right shows the probe combinations loaded in each lane. 20P, 20-nucleotide promoter; 13P, 13-nucleotide promoter; CT, cytosine-thymine.





**Figure S2.** (A) Agarose (2.5%) gel electrophoresis confirming hybridization of the transcription template strand with A flap probes. The table on the right shows the probe combinations used in each lane. (B) Real-time fluorescence monitoring of A flap probes. (C) Agarose gel electrophoresis analysis of T flap probes. (D) Real-time fluorescence monitoring of T flap probes. (E) Agarose gel electrophoresis analysis of C flap probes. (F) Real-time fluorescence monitoring of C flap probes. (G) Agarose gel electrophoresis analysis of G flap probes. (H) Real-time fluorescence monitoring of G flap probes. The table on the right shows the probe combinations used in each lane. A, adenine; T, thymine; G, guanine; C, cytosine; 13P, 13-nucleotide promoter; NPC, non-promoter control.


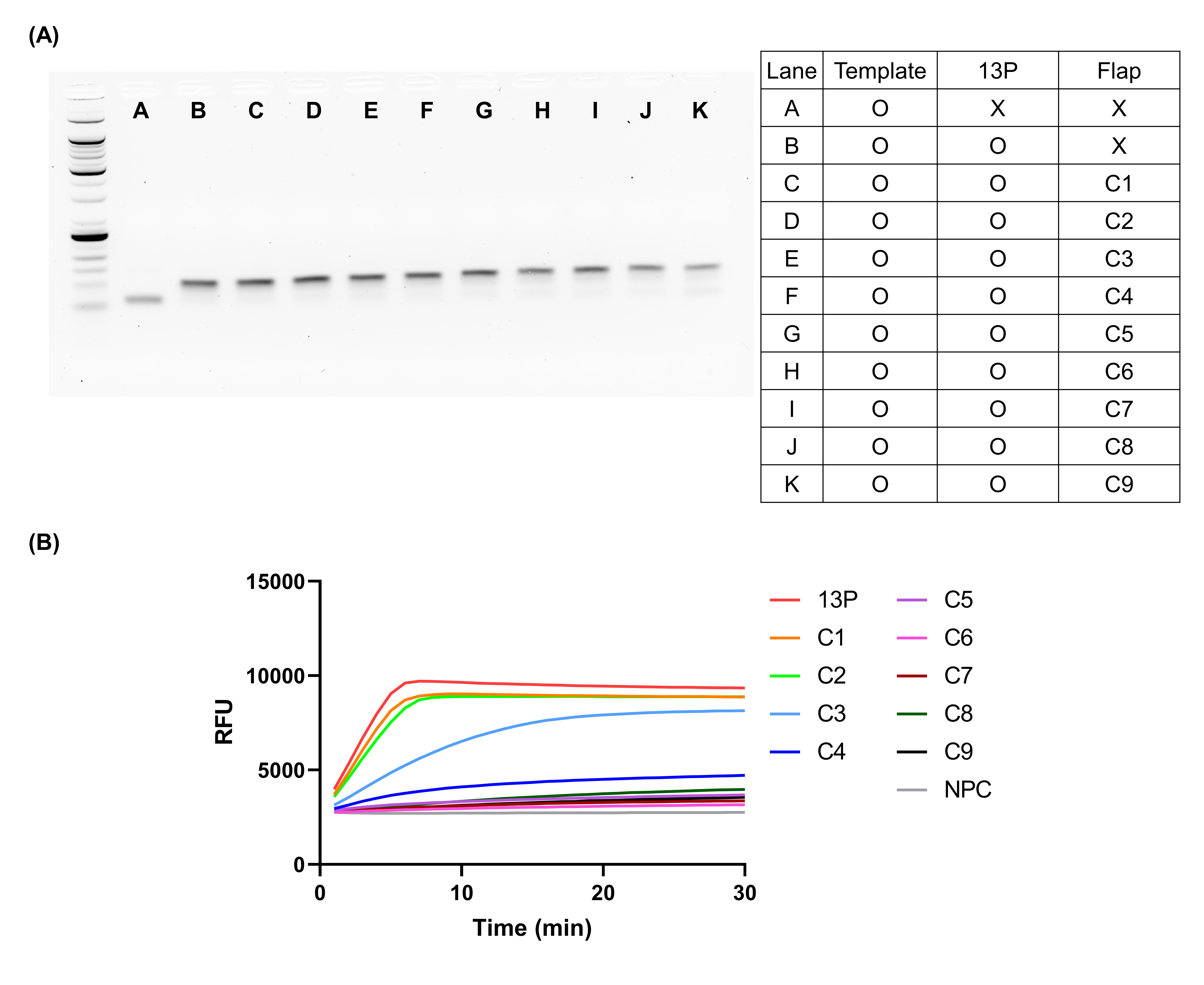


**Figure S3.** (A) Agarose (2.5%) gel electrophoresis confirming hybridization of the transcription template strand with C flap probes (C1–C9). The table on the right shows the probe combinations used in each lane. (B) Real-time fluorescence monitoring of C flap probes. 13P, 13-nucleotide promoter; C, cytosine; NPC, non-promoter control.


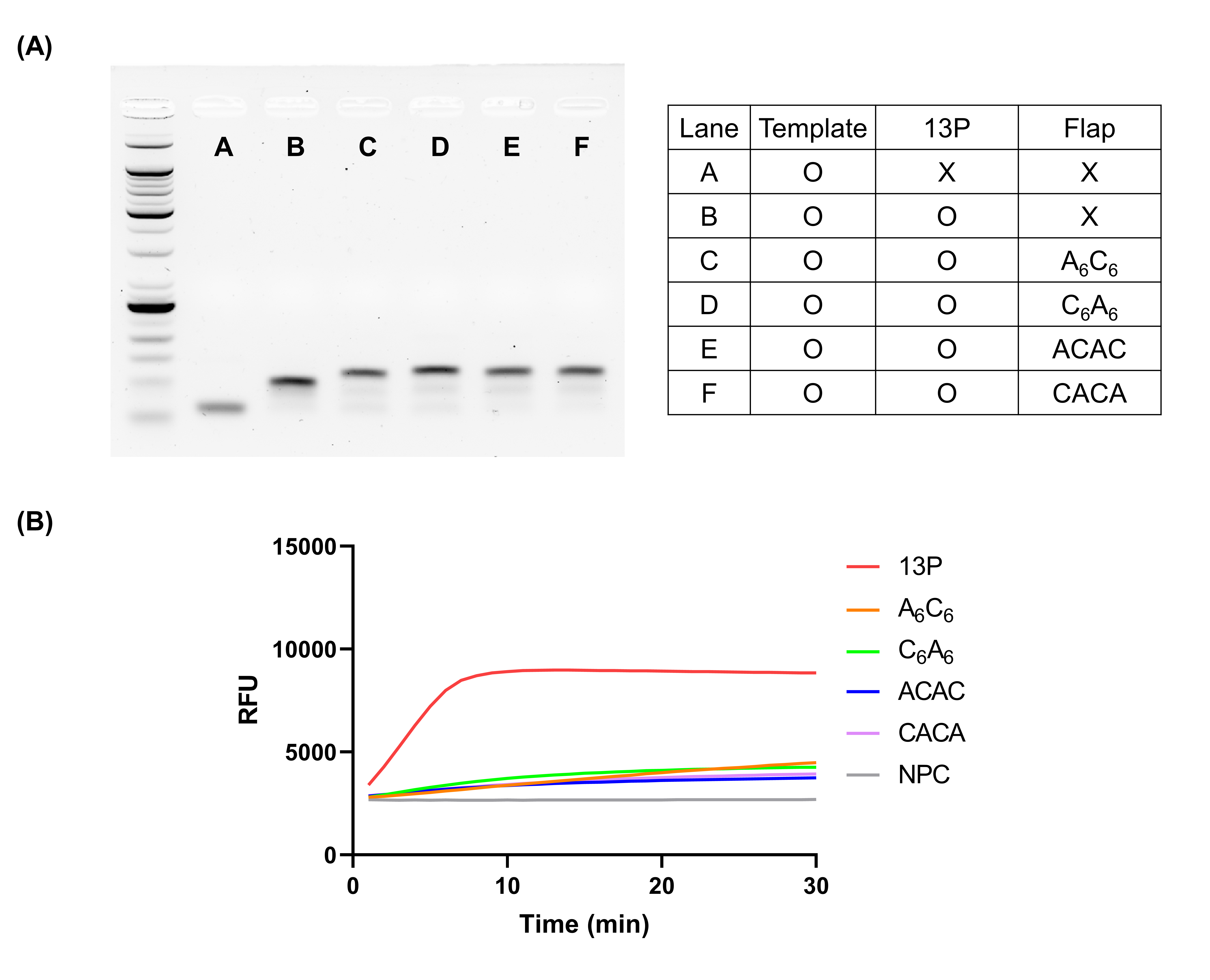


**Figure S4.** (A) Agarose (2.5%) gel electrophoresis confirming hybridization of the transcription template strand with flap probes composed of A and C. The table on the right shows the probe combinations used in each lane. (B) Real-time fluorescence monitoring of flap probes composed of A and C. 13P, 13-nucleotide promoter; A, adenine; C, cytosine; NPC, non-promoter control.


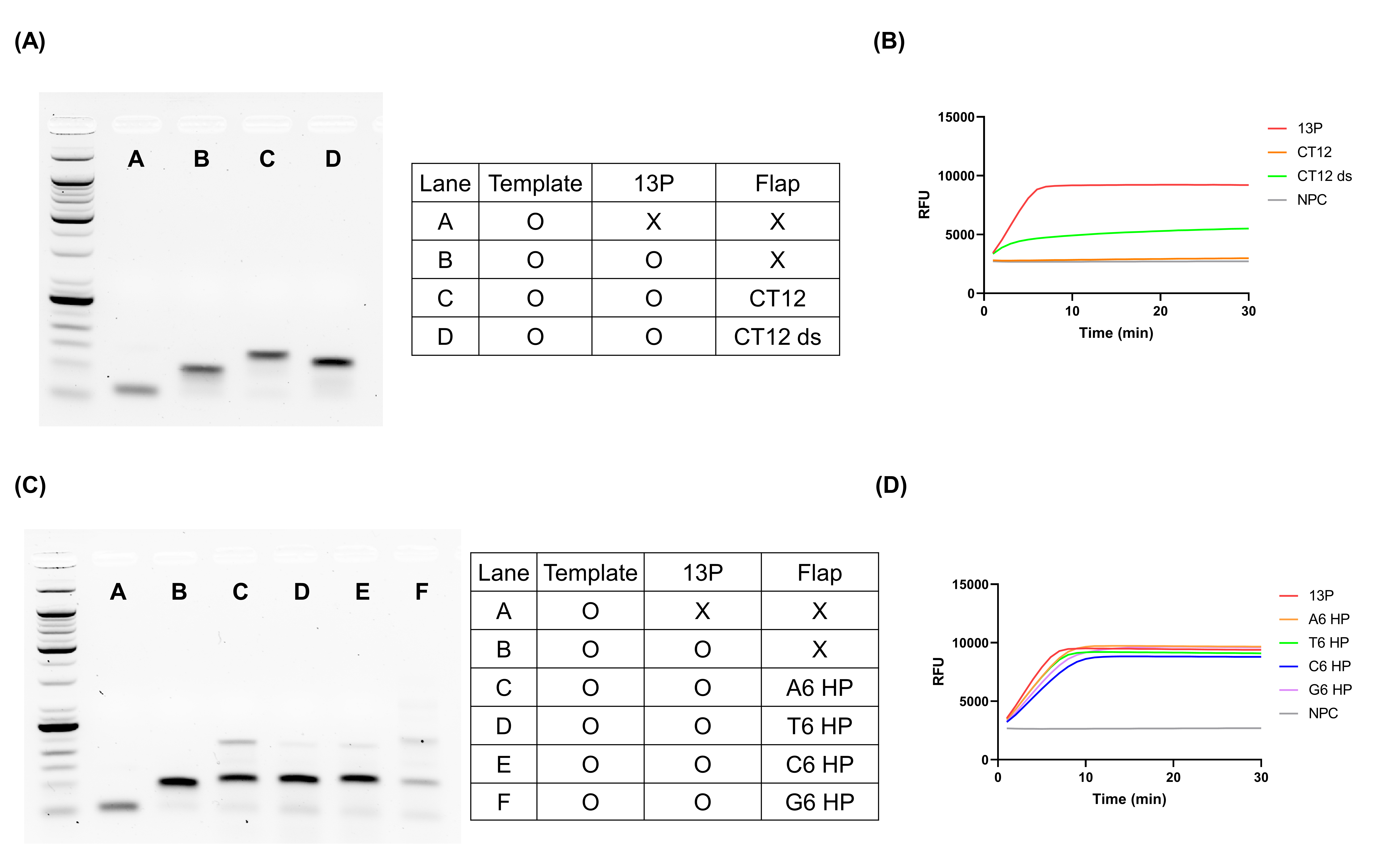


**Figure S5*.*** (A) Agarose (2.5%) gel electrophoresis confirmed the hybridization of the transcription template strand with CT12 and CT12 ds (double-stranded) flap probes. The table on the right shows the probe combinations used in each lane. (B) Real-time transcription monitoring of CT12 and CT12 ds flap probes. (C) Agarose gel electrophoresis confirmed the hybridization of the transcription template strand with hairpin (HP) flap probes. The table on the right shows the probe combinations used in each lane. (D) Real-time transcription monitoring of HP flap probes. A, adenine; T, thymine; C, cytosine; G, guanine; 13P, 13-nucleotide promoter; NPC, non-promoter control.


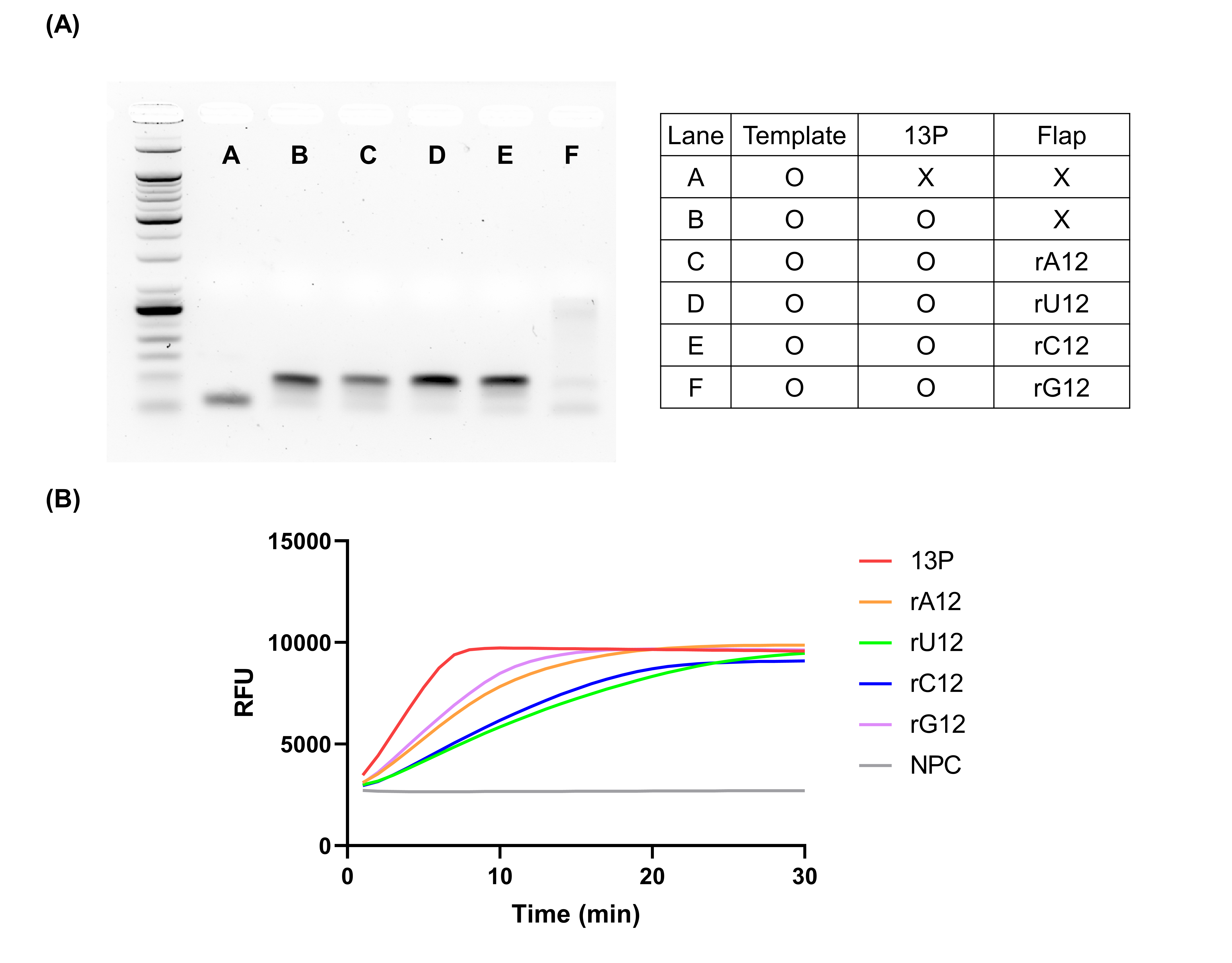


**Figure S6*.*** (A) Agarose (2.5%) gel electrophoresis confirmed the hybridization of the transcription template strand with ribonucleoside triphosphate (rNTP) flap probes. The table on the right shows the probe combinations used in each lane. (B) Real-time transcription monitoring of rNTP flap probes. A, adenine; U, uracil; C, cytosine; G, guanine; 13P, 13-nucleotide promoter; NPC, non-promoter control.


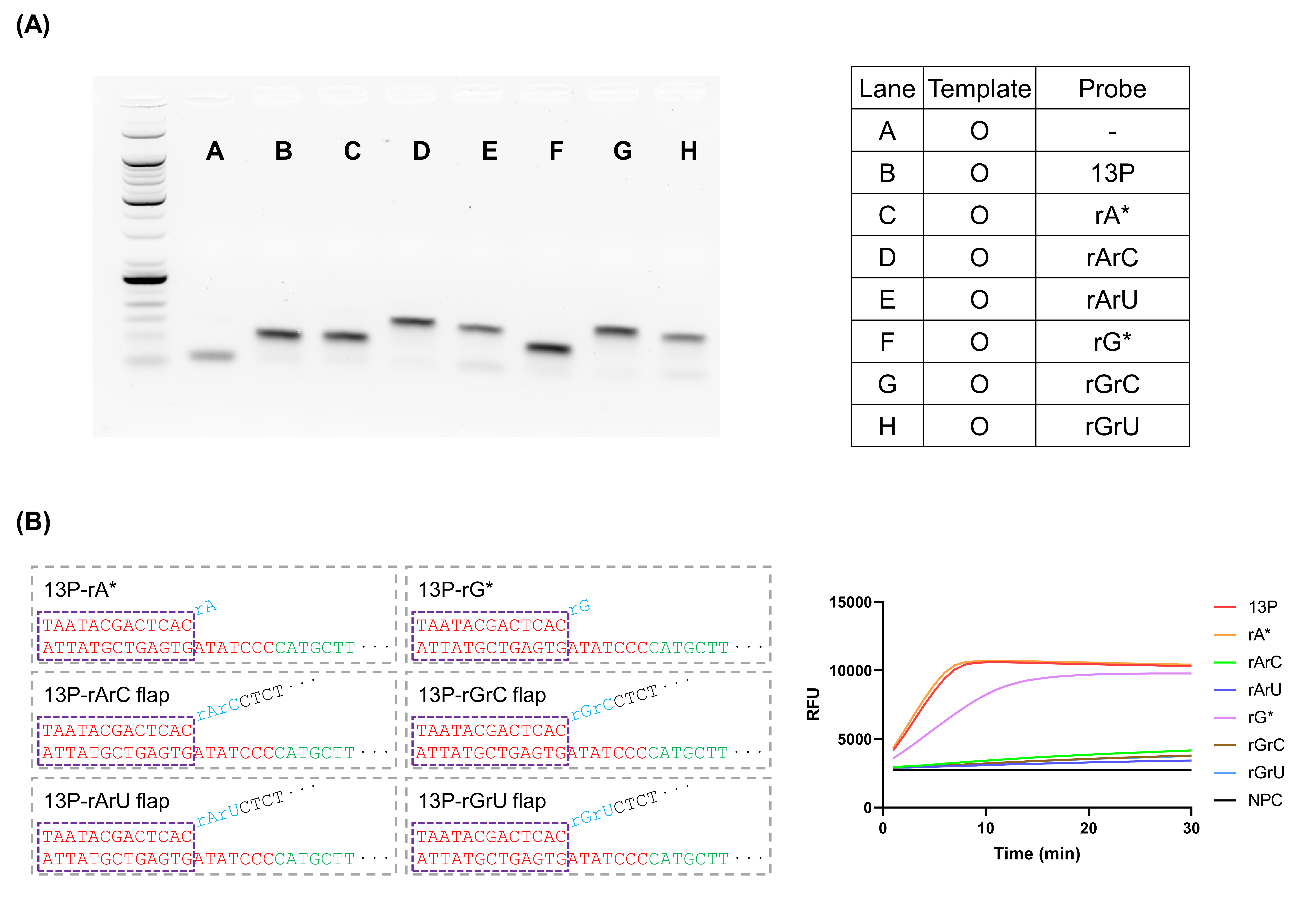


**Figure S7*.*** (A) Agarose (2.5%) gel electrophoresis confirmed the hybridization of the transcription template strand with DNAzyme flap probes. The table on the right shows the probe combinations used in each lane. (B) Real-time transcription monitoring of DNAzyme flap probes. The grey dotted boxes indicate the sequence of each DNAzyme flap promoter structure, while the violet dotted boxes indicate the hybridized promoter region. The red, blue and green sequences indicate the 13P region, the RY (R: rA and rG; Y: rC and rU) region, and the transcribed sequence, respectively. “*” indicates a promoter sequence without a CT flap. A, adenine; T, thymine; C, cytosine; G, guanine; U, uracil; 13P, 13-nucleotide promoter; NPC, non-promoter control.


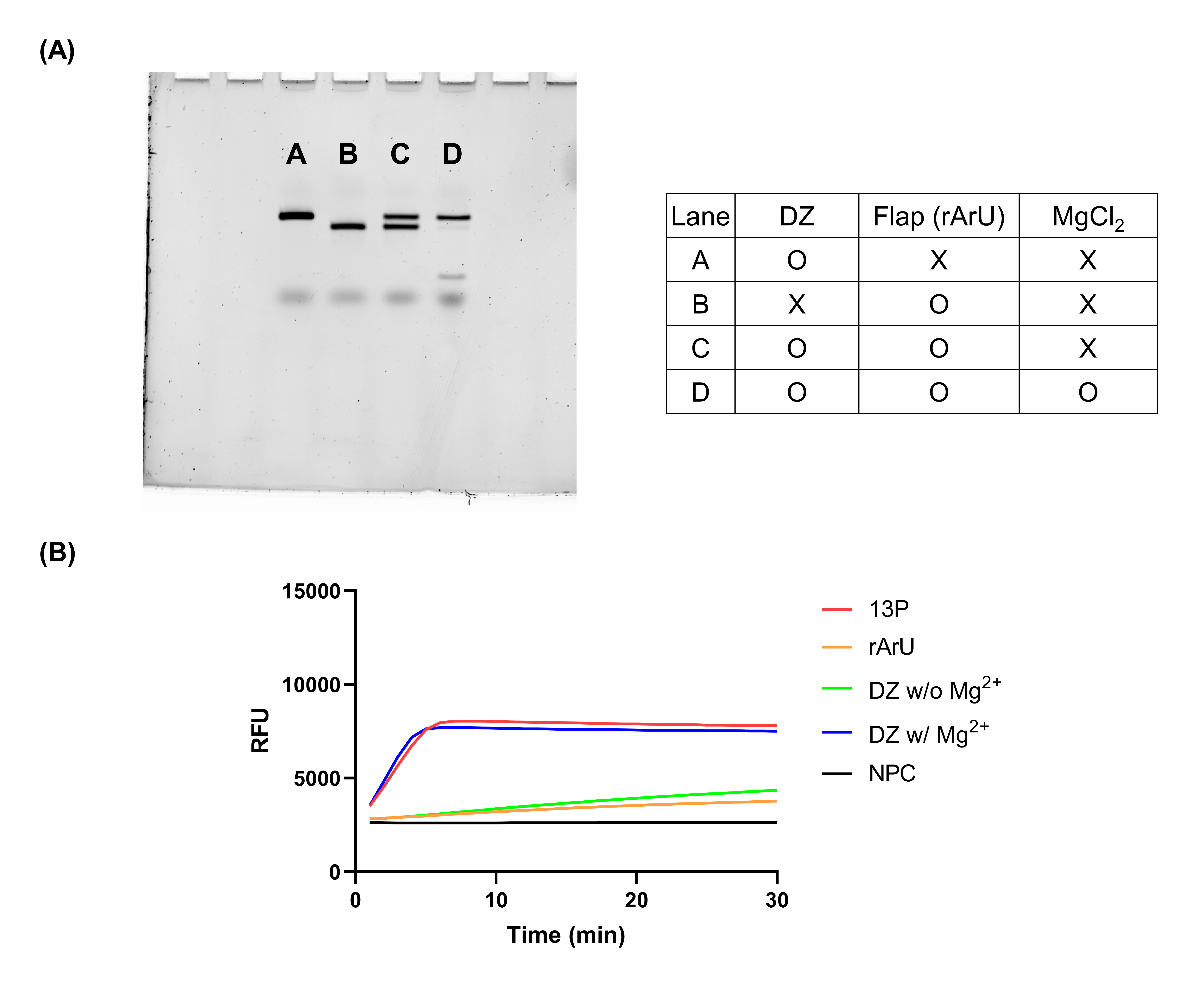


**Figure S8.** (A) Denaturing urea-PAGE analysis confirmed the cleavage of the rArU flap by DNAzyme (DZ) in the presence of Mg^2+^. The table below the gel image shows the probe combinations used in each lane. (B) Real-time transcription monitoring of DNAzyme flap probes. A, adenine; U, uracil; 13P, 13-nucleotide promoter; NPC, non-promoter control.


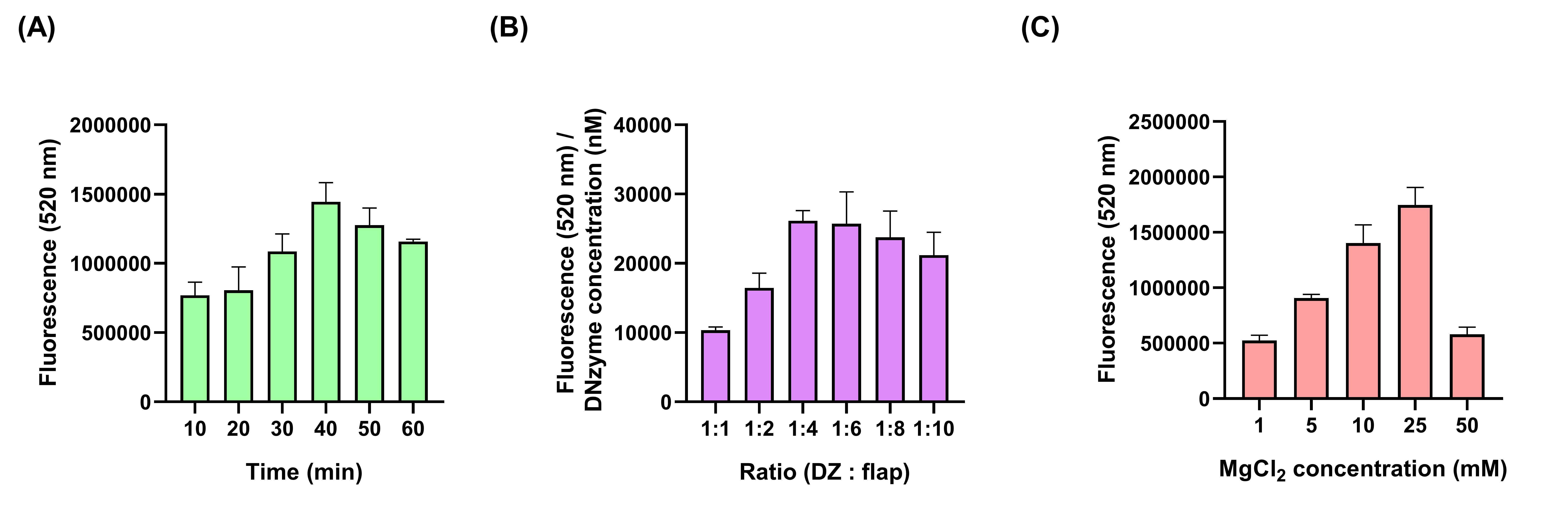


**Figure S9.** Optimization of DNAzyme-mediated flap promoter induced transcription control (D-FIT) system. (A) Optimization of DNAzyme (DZ) reaction time. (B) Optimization of ratio between DZ and flap probes. (C) Optimization of MgCl_2_ concentration. Data represent mean ± s.d. (n = 3).

**
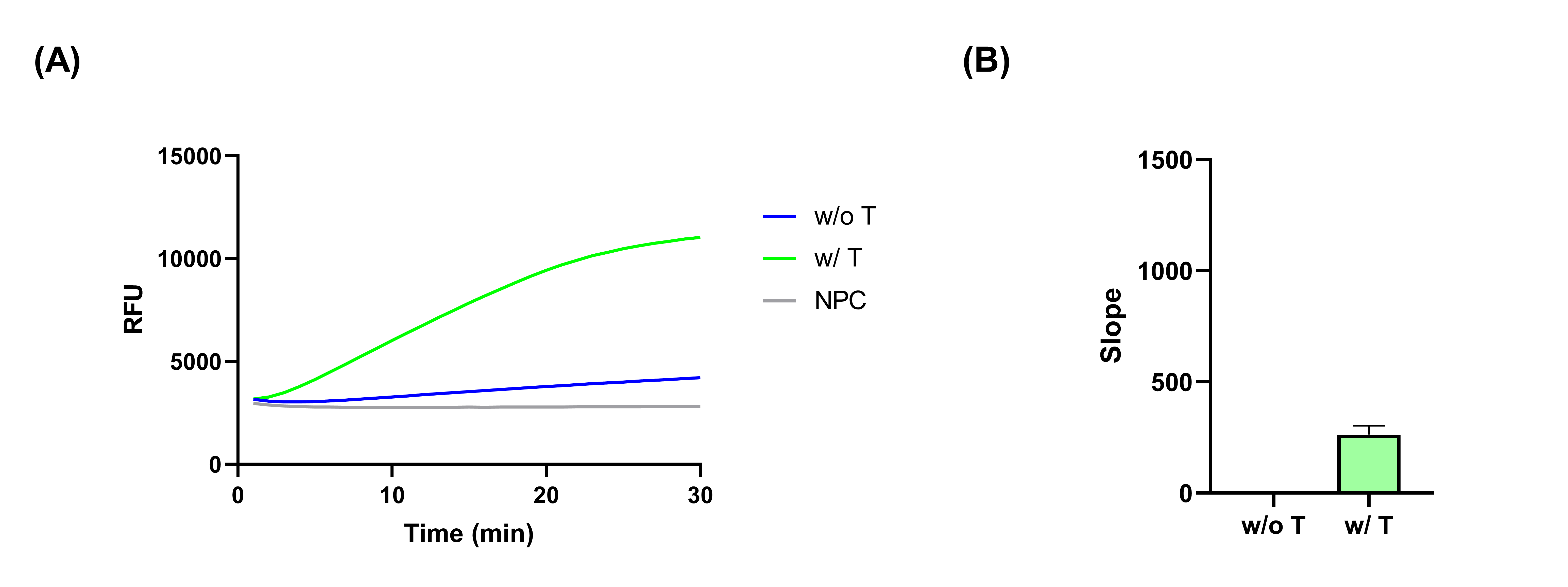
**

**Figure S10.** (A) Real-time transcription monitoring of MNAzyme-mediated flap promoter induced transcription control (M-FIT) system. (B) Transcriptional activity analysis of M-FIT system. NPC, non-promoter control; T, trigger DNA. Data represent mean ± s.d. (n = 3).
